# High Oleic Acid Peanut Oil and Extra Virgin Olive Oil Supplementation Attenuate Metabolic Syndrome in Rats by Modulating the Gut Microbiota

**DOI:** 10.3390/nu11123005

**Published:** 2019-12-07

**Authors:** Zhihao Zhao, Aimin Shi, Qiang Wang, Jinrong Zhou

**Affiliations:** 1 Institute of Food Science and Technology, Chinese Academy of Agricultural Sciences/Key Laboratory of Agro-Products Processing, Ministry of Agriculture and Rural Affairs, Beijing 100193, China; zhaozhihao1991@163.com (Z.Z.); sam_0912@163.com (A.S.); 2 Nutrition/Metabolism Laboratory, Beth Israel Deaconess Medical Center, Harvard Medical School, 330 Brookline Avenue, Boston, MA 02215, USA; jrzhou@bidmc.harvard.edu

**Keywords:** extra virgin olive oil, high oleic acid peanut oil, metabolic syndrome, gut microbiota, high-fructose-high-fat diet

## Abstract

Unhealthy dietary patterns are important risk factors for metabolic syndrome (MS), which is associated with gut microbiota disorder. High oleic acid peanut oil (HOPO) and extra virgin olive oil (EVOO), considered as healthy dietary oil, are rich in oleic acid and bioactive phytochemicals, yet efficacy of MS prevention and mechanisms linking to gut microbiota remain obscure. Herein, we investigated HOPO and EVOO supplementation in attenuating diet-induced MS, and the potential mechanisms focusing on modulation of gut microbiota. Physiological, histological and biochemical parameters and gut microbiota profiles were compared among four groups fed respectively with the following diets for 12 weeks: normal chow diet with ordinary drinking water, high-fat diet with fructose drinking water, HOPO diet with fructose drinking water, and EVOO diet with fructose drinking water. HOPO or EVOO supplementation exhibit significant lower body weight gain, homeostasis model assessment-insulin resistance (HOMA-IR), and reduced liver steatosis. HOPO significantly reduced cholesterol (TC), triglyceride (TG), and low-density lipoprotein (LDL) level, while EVOO reduced these levels without significant difference. HOPO and EVOO prevented gut disorder and significantly increased *β*-diversity and abundance of *Bifidobacterium*. Moreover, HOPO significantly decreased abundance of *Lachnospiraceae* and *Blautia*. These findings suggest that both HOPO and EVOO can attenuate diet-induced MS, associated with modulating gut microbiota.

## 1. Introduction

Metabolic syndrome (MS), characterized by three or more of five medical components (including obesity, hyperglycemia, dyslipidemia, hypertension, and insulin resistance), can enhance the risk of type 2 diabetes (T2DM) and cardiovascular disease (CVD) [1]. Compared with people without MS, people suffering from MS are twice as likely to die and three times as likely to have a heart attack or stroke. The prevalence of MS is growing worldwide, especially in developing countries, which is mainly due to the changes in lifestyles and dietary patterns. It is estimated that about one in four people worldwide suffer from MS [2]. The current national prevalence of MS among Chinese adults is 24.2 %, which is a sharp growth compared with the value of 9.8% calculated 10 years ago under the same standard of diagnosis [3,4]. Thus, prevalence of MS has become a severe threat to modern society, and its preventative strategies are significant.

A large amount of data indicates that gut microbiota is essential for maintaining the metabolic homeostasis of the host [5,6]. A wide variety of commensal microbes colonizes gut lumen. For example, the population quantity of microbes in the gut microbiota is approximately 10 times that of somatic cells in human body. These microbes participate in most metabolic activities in vivo. Recently, some beneficial species or genera of gut microbiota have been demonstrated to be negatively associated with the development of MS, such as *Akkermansia* [7], *Bifidobacterium* [5], and *Lactobacillus* [8]. In contrast, over-proliferation of some pro-inflammatory or pathogenic gut microbiota, such as *Erysipelotrichaceae*, *Coriobacteriaceae*, and *Streptococcaceae*, are associated with the development of obesity, systemic inflammation, and metabolic disorders in both humans and rodents [8,9,10]. Thus, regulating diet-induced gut microbiota disruption has been presented as a potential intervention target for the prevention of MS and related diseases [11].

While many potential risk factors for MS have been identified, dietary patterns definitely play an important role. The intake and profile of dietary fat plays an accepted role in CVD risk and the development of cardio-metabolic diseases such as those syndromes included in the diagnostic criteria of MS [12,13]. It has been demonstrated that dietary fat intake is a dominant factor influencing the pattern of gut microbiota [6,14], and the *Firmicutes*/*Bacteroidetes* ratio (F/B ratio) [15]. The F/B ratio is positively correlated with lean phenotype/weight loss, which has been supported by several studies [16]. Compared with diets enriched with saturated fatty acids, diets with high levels of unsaturated fatty acids have been associated with lower body weight gain, and lipid accumulation in liver. These effects have been confirmed to be related to the diet-induced changes of gut microbiota [17]. For the past few years, dietary fructose intake has also come under scrutiny. Several epidemiologic surveys suggest an underlying link between consumption of fructose containing sugars such as high fructose corn syrup, and sucrose and risk factors for CVD, and diagnostic standards of MS [18,19,20]. A meta-analysis including 15 studies also showed that fructose consumption from processed foods is one of the causes of some chronic disorders such as MS among healthy adults [21]. The effect of fructose on development of MS is driving hepatic fat, which can induce insulin resistance [22]. The specific effects of fructose on liver are particularly related to a vicious circle that starts with liver steatosis driving insulin resistance. Fructose derived advance glycation end-products may promotes inflammation by engaging receptor for advanced glycation end products (RAGE). Thus, rats or mice fed with a high-fructose-high-fat diet (HFHFD) are used as an animal model of MS.

Virgin olive oil is the main source of dietary fat at the core of Mediterranean diet. There is a widespread recognition on association between the regular consumption of virgin olive oil and a lower risk of MS [23]. These beneficial biological activities have been attributed not only to the high monounsaturated fatty acid (MUFA) content but also to the minor bioactive phytochemicals [24]. Recent research indicates the possibility that virgin olive oil may attenuate MS associated with modulation of intestinal microbiota [25]. Similarly, HOPO is also rich in MUFA (providing up to 80% of the fatty acid composition, similar even higher level to olive oil) and minor bioactive phytochemicals, such as polyphenol, phytosterols, and vitamin E, etc. However, MS prevention and gut microbiota modulating of HOPO has never been studied.

Hence, in this study, we conducted a comparison of the effects of HOPO, and EVOO supplement on MS in HFHFD-fed rats. Then, to illustrate the possible mechanisms, the profile of the gut microbiota was analyzed by utilizing a 16S rRNA sequencing technique, and the biochemical indexes were also determined. We demonstrated that both HOPO and EVOO can attenuate the HFHFD-induced MS. Moreover, this study presents a new perception of gut microbiota modulation in the prevention of MS by dietary fats rich in MUFA.

## 2. Materials and Methods

### 2.1. Materials

HOPO provided by Luhua Group (Laiyang, China). EVOO purchased from Mueloliva Co. Ltd. (Córdoba, Spain). Fatty acid profiles of HOPO, and EVOO are shown in Table S1. Fructose purchased from SIWANG SUGAR Co. Ltd. (Binzhou, China).

### 2.2. Animals and Treatment

The 48 6-week-old male SD rats were purchased from Beijing Vital River Laboratory Animal Technology Co., Ltd. (Beijing, China). After fed adaptively for 1 week, rats were randomly divided into 4 groups (n = 12) to receive respectively the following diets ad libitum for 12 weeks: (A) NC (normal control, normal chow diet + ordinary drinking water); (B) M (model, high-fat diet + drinking water contains 10% fructose); (C) HOPO (high oleic acid peanut oil diet, high-fat diet contains 10% HOPO + drinking water contains 10% fructose); and (D) EVOO (extra virgin olive oil diet, high-fat diet contains 10% EVOO + drinking water contains 10% fructose). Compositions of the diets are shown in Table S2. All the diets were purchased from Trophic Animal Feed High-Tech Co. Ltd. (Nantong, China). The rats were kept in a well-ventilated room maintained at 23 ± 2 °C with 12-h light-dark cycles. Their body weight and food and water intake were recorded weekly. Fasting blood-glucose, oral glucose tolerance test (OGTT), and insulin tolerance test (ITT) were measured at 0, 4, 8 and 12 weeks. Protocols for animal studies were approved by the Institutional Animal Care and Use Committee of Beijing Vital River Laboratory Animal Technology Co., Ltd. (VR IACUC, Beijing, China, No. P2018036).

### 2.3. Insulin Resistance Assessment

Oral glucose tolerance test (OGTT): the rats were fasted for 12-h and orally infused with glucose (2 g/kg). The blood was collected from tail vein and its glucose level was measured with glucometer (Johnson and Johnson Investment, Co. Ltd., Shanghai, China) before (0 min) and after gavage (15, 30, 60, 90 and 120 min). The area under curve (AUC) was calculated to represent the glucose tolerance. AUC was calculated using the formula AUC = 0.25 × (G0 + G15)/2 + 0.25 × (G15 + G30)/2 + 0.5 × (G30 + G60)/2 + 0.5 × (G60 + G90)/2 + 0.5 × (G90 + G120)/2, where G0, G15, G30, G60, G90, and G120 were blood glucose levels at different time points.

Insulin tolerance test (ITT): the rats were fasted for 12-h and received intraperitoneal injection of insulin solution (0.75 U/kg). Blood glucose test and AUC value calculation were carried out as for OGTT. HOMA-IR was calculated using the formula HOMA-IR = (FPG × FINS)/22.5, where FPG and FINS were fasting blood-glucose level and fasting insulin level.

### 2.4. Serum Biochemical Analysis

At the end of experiment, the animals were sacrificed, and blood samples were collected. Serum samples were prepared by centrifugation (4 °C, 2000× *g* for 15 min). SerumTG, TC, low density lipoprotein (LDL), high density lipoprotein (HDL), insulin, free fatty acid (FFA), and TNF-a levels were analyzed by using kits from Nanjing Jiancheng Bioengineering Institute (Nanjing, China).

### 2.5. Histopathological Examination and TG Level in Liver Tissue

Freshly isolated liver tissues were embedded in optimal cutting temperature (OCT) compound and stored at −80 °C ultra-cold storage freezer after flash frozen. The liver tissues were cut into 8 μm sections on a cryostat, and fixed with 4% paraformaldehyde for 10 min. The sections were stained with oil red O for 12 min and re-dyed with hematoxylin for 4 min. The sections were observed under a light microscope (Primo star, Carl Zeiss Microscopy GmbH, Jena, Germany). TG level in liver tissue were analyzed by using kits from Nanjing Jiancheng Bioengineering Institute (Nanjing, China).

### 2.6. Gut Microbiota Analysis

After 12 weeks of experimental treatment, fresh fecal samples were collected in sterilized Eppendorf tubes and stored at −80 °C ultra-cold storage freezer after flash frozen. Ten rats were selected randomly from each group for gut microbiota analysis. According to the instructions, QIAamp DNA stool Mini Kit from Qiagen (Hilden, Germany) was used to extract bacterial genomic DNA from frozen fecal samples temporary stored at −80 °C for 24 h. The 16S rRNA gene comprising V3 and V4 regions was magnified by PCR using composite specific bacterial primers (Table S3). Thermal cycling was as following: 95 °C for 5 min (1 cycle), 95 °C for 30 s/50 °C for 30 s/72 °C for 40 s (25 cycles), and a final extension at 72 °C for 7 min. High-throughput pyrosequencing of the PCR products was performed on an Illumina MiSeq platform by Biomarker Technologies Co, Ltd. (Beijing, China).

The raw-paired end reads from the original DNA fragments were merged using FLASH and assigned to each sample according to the unique barcodes. High-quality reads for bioinformatics analysis were performed, and all of the effective reads from each sample were clustered into operational taxonomic units (OTUs) based on a 97% sequence similarity according to UCLUST. After the OTU data are normalized (logarithm), the top 80 species are selected and drawn based on the R heatmap. For alpha diversity analysis, we rarified the OTUs to several metrics, including curves of OTU rank, rarefaction and Shannon, and calculated indexes of Shannon, Chao1, Simpson, and ACE. For *β*-diversity analysis, heatmap of RDA-identified key OTUs, principal component analysis (PCA) and nonmetric multidimensional scaling (NMDS) were performed using QIIME. The LDA effect size (LEfSe) analysis was performed for the quantitative analysis of biomarkers among each group. Briefly, LEfSe analysis, LDA threshold of >4, used the nonparametric factorial Kruskal-Wallis (KW) sum-rank test and then used the (unpaired) Wilcoxon rank-sum test to identify the most differently abundant taxa. Metastats analysis obtained *p* values by T-test of relative abundance data. The species causing the difference in the composition of the two groups were screened out by *p* values (*p* < 0.05).

### 2.7. Statistical Analysis

Results were expressed as means ± SD. The statistical analysis was performed using SPSS, version 20 (IBM, Armonk, NY, USA). Differences between groups were statistically analyzed using ANOVA followed by Duncan’s test and considered statistically with a level of *p* < 0.05.

## 3. Results

### 3.1. Body Weight, Body Weight Gain, Energy Intake and Energy Efficiency

**Figure 1:**
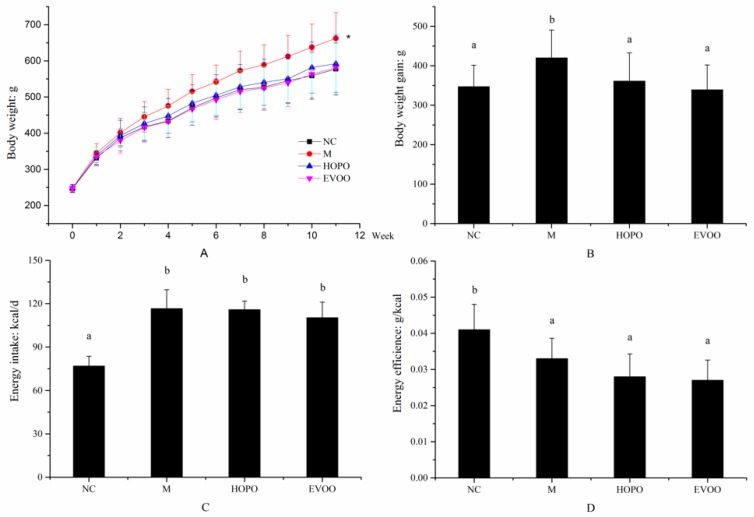
Effects of treatments on body weight, body weight gain, energy intake and energy efficiency. Body weight (**A**), body weight gain (**B**), energy intake (**C**) and energy efficiency, calculated as body weight gain/energy intake (**D**). Data are expressed as means ± SD (*n* = 12 for each group). The different letters represent significant differences between different groups (*p* < 0.05). * *p* < 0.05 vs. NC, HOPO, and EVOO. NC: normal control group, M: model group, HOPO: high-oleic acid peanut oil group, EVOO: extra virgin olive oil group.

As shown in Figure 1A,B, at 12-week, body weight and body weight gain of the M group was significantly higher than other groups (*p* < 0.05). The M group showed a 420.33 ± 70.44 g body weight gain, while body weight gain of NC, HOPO, and EVOO group was 347.25 ± 54.45 g, 360.501 ± 71.86 g, and 339.25 ± 63.24 g, respectively. There was no significant difference between the NC, HOPO, and EVOO group. In order to find out whether the effect of HOPO or EVOO was through regulating the appetite or energy intake of the rats, energy intake was determined. There were no significant differences of energy intake among M, HOPO, and EVOO groups (Figure 1C). The energy efficiency of NC group was significantly higher than HFHFD groups (NC group 0.041 ± 0.0070 g/kcal vs. M, HOPO, and EVOO group 0.033 ± 0.0056, 0.028 ± 0.0063, and 0.027 ± 0.0056 g/kcal, *p* < 0.05, Figure 1D), whereas it was not significantly different among the HFHFD groups. The data suggest that the protective effect of HOPO and EVOO supplementation against HFHFD-induced weight gain was not through suppression of appetite or energy intake.

### 3.2. Histopathological Examination and TG Level in Liver Tissue

**Figure 2:**
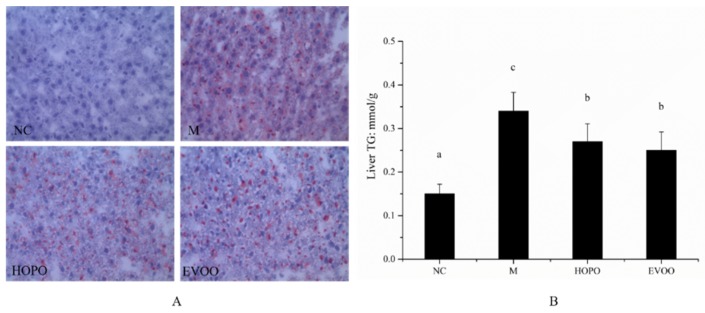
Lipid staining of the liver section and TG level in liver. oil red O staining liver section, magnification 400 × (**A**), TG content in liver (**B**). Data are expressed as means ± SD, *n* = 12 for each group and the different letters represent significant differences between different groups (for B, *p* < 0.05). NC: normal control group, M: model group, HOPO: high-oleic acid peanut oil group, EVOO: extra virgin olive oil group.

High fat diet is known to cause hepatic inflammation, injury, and adiposis. To evaluate liver histological changes, liver tissues were stained with oil red O. As shown in Figure 2A, the lipid droplets in the liver cells are red and cell nucleus are blue in oil red O staining section. Liver tissues in NC group showed no significant red droplet or areas. Compare with NC group, liver tissues in M group showed more fat accumulation. However, compare with M group, liver tissues in HOPO and EVOO group showed a less fat accumulation. This trend was also in line with the result of TG level in liver. As shown in Figure 2B, Liver TG levels in HOPO and EVOO group were significantly lower than M group. HOPO and EVOO supplementation dramatically reduced the degree of HFHFD-induced adiposis hepatica in MS rats.

### 3.3. Biomarkers of Blood Glucose Level and Insulin Resistance

As shown in Table 1, HFHFD induced a higher fasting blood-glucose level. At 12-week, fasting blood-glucose level in NC group was significantly lower than M, HOPO, and EVOO group (NC group 4.38 ± 0.42 mmol/L vs M, HOPO, and EVOO group 6.08 ± 0.76, 6.53 ± 0.65, and 5.79 ± 0.56 mmol/L, *p* < 0.05), and there was no significant difference between the M, HOPO, and EVOO group. At 12-week, HFHFD induced a higher AUC value of OGTT in M, HOPO, and EVOO group than NC group, but there was no significant difference between them. For ITT, AUC value in M group were significantly higher than that in other groups after 8 and 12 weeks of treatment, which indicates that rats in HOPO and EVOO group had higher insulin sensitivity than M group. Consistent with ITT results, serum insulin and HOMA-IR of M group were significantly higher than NC group (M group 1.70 ± 0.33 ug/L and 10.88 ± 1.71 vs. NC group 0.89 ± 0.12 ug/L and 4.15 ± 0.73, *p* < 0.05), and HOPO or EVOO supplementation resulted in a significant reduction of fasting insulin levels and HOMA-IR (HOPO group 1.19 ± 0.21 ug/L and 8.67 ± 2.02; EVOO group 1.36 ± 0.26 ug/L and 8.43 ± 1.86, *p* < 0.05). The above results suggest that HOPO and EVOO supplementation could prevent the insulin resistance in HFHFD-induced MS rats.

### 3.4. Biomarkers of Metabolic Syndrome in Serum

As depicted in Table 2, TC, TG, and LDL levels of M group were significantly higher than NC group (M group 3.62 ± 0.50, 5.99 ± 2.09, and 0.56 ± 0.21 mmol/L vs. NC group 3.00 ± 0.79, 1.95 ± 0.42, and 0.40 ± 0.14 mmol/L, *p* < 0.05), while HDL level of M group was lower than NC group without significant difference. HOPO significantly reduced TC, TG, and LDL level than M group (HOPO group: 3.06 ± 0.65, 4.23 ± 1.45, and 0.38 ± 0.13 mmol/L, *p* < 0.05). In contrast, TC, TG, and LDL level of EVOO group were decreased non-significantly (EVOO group 3.18 ± 0.40, 5.79 ± 2.22, and 0.50 ± 0.18 mmol/L, *p* > 0.05). For HDL level, there were no significant differences among any groups (NC, M, HOPO, and EVOO group: 0.84 ± 0.15, 0.71 ± 0.25, 0.69 ± 0.23, and 0.76 ± 0.25, respectively, *p* > 0.05). For HDL/LDL ratio, both HOPO and EVOO group increased than M group, but less than NC group (NC, M, HOPO, and EVOO group: 2.10 ± 0.25, 1.27 ± 0.12, 1.82 ± 0.18, and 1.52 ± 0.21, respectively, *p* > 0.05). Compared with NC group, M group showed a significant increase of free fatty acid and TNF-a level (M group 555.80 ± 55.34 umol/L and 257.74 ± 75.73 pg/mL vs. NC group 459.96 ± 38.76 umol/L and 196.11 ± 34.67 pg/mL, *p* < 0.05). HOPO and EVOO supplementation remitted the increased amplitude, but there was no significant difference with M group (HOPO group 526.80 ± 60.18 umol/L and 208.16 ± 58.73 pg/mL; EVOO group 512.06 ± 47.15 umol/L and 220.35 ± 63.03 pg/mL, *p* > 0.05). These results imply that both HOPO and EVOO supplementation can improve serum lipids profile in HFHFD-induced MS rats, and protective effects of HOPO are better than EVOO.

### 3.5. Overall Structural Changes of Gut Microbiota

**Figure 3:**
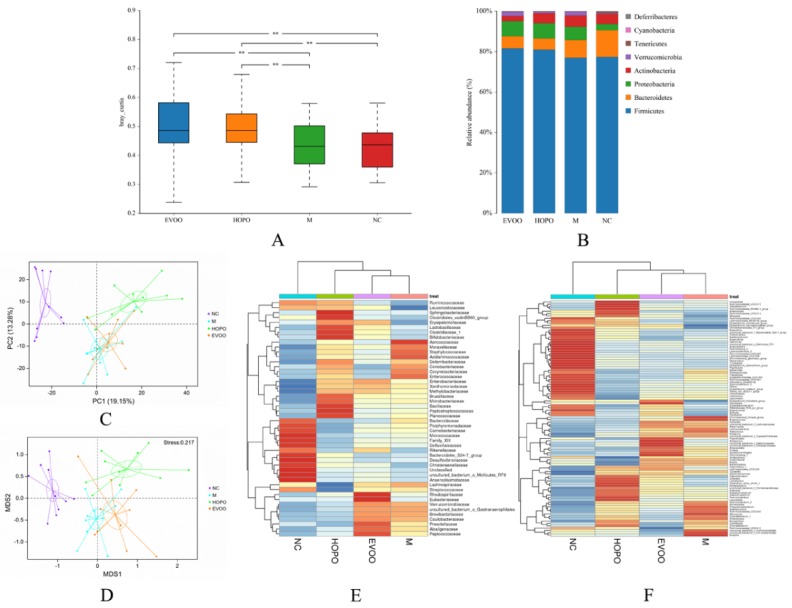
The results of the β-diversity, relative abundance at phylum levels, principal component analysis (PCA), nonmetric multidimensional scaling (NMDS) analysis (based on bray curtis) and relative abundance heatmap (*n* = 10 for each group). β-diversity (**A**), relative abundance at phylum levels (**B**), PCA(**C**), NMDS (**D**), family heatmap based on groups (**E**) and genus heatmap based on groups (**F**). ** *p* < 0.01 between two groups. NC: normal control group, M: model group, HOPO: high-oleic acid peanut oil group, EVOO: extra virgin olive oil group.3.6. Key Phylotypes in Response to HOPO and EVOO Supplementation.

In all detected OTUs, 386 were shared by all groups (Figure S1). The unique OTUs were 3, 0, 0, and 3 in NC, M, HOPO, and EVOO group, respectively. There was no significant difference in the value of Simpson and Shannon among the four groups (Table S4). Significant differences of *β*-diversity distance were found between NC, M, HOPO, and EVOO group (Figure 3A). HOPO and EVOO significantly increased *β*-diversity of gut microbiota composition. At phyla level, *Firmicutes*, *Bacteroidetes*, and *Actinobacteria* were the dominant phyla in all groups (Figure 3B). The F/B ratios of three HFHFD groups were higher than NC group, and HOPO and EVOO group were higher than M group, even though obesity and serum parameters attenuated by HOPO and EVOO supplementation (NC, M, HOPO, and EVOO group: 5.65, 9.41, 14.39, and 12.91, respectively, Figure 3B). Analyses of PCA, NMDS, and heatmaps showed the similarity degrees of gut microbiota in four groups. The scatter diagrams of PCA and NMDS showed that points of four groups were clearly differentiated, in particularly between NC and three HFHFD groups (Figure 3C,D). The heatmaps showed that HOPO, EVOO, and NC were more similar than NC to M, and HOPO was more similar with NC than EVOO (Figure 3E,F). This trend was also in line with the scatter diagrams of PCA and NMDS. Taken together, HOPO and EVOO supplementation attenuate the imbalance of gut microbiota induced by HFHFD.

**Figure 4:**
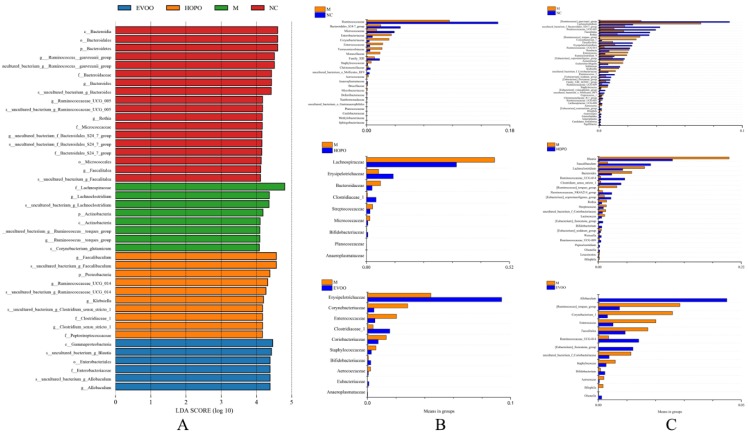
The results of linear discriminant analysis (LDA) effect size (LEfSe) analysis and gut microbiota at family and genus level with significant difference (*n* = 10 for each group). LEfSe analysis (**A**), Family level (**B**) and genus (**C**) with significant difference. At genus level, only the species that relative abundance higher than 0.1% were listed. NC: normal control group, M: model group, HOPO: high-oleic acid peanut oil group, EVOO: extra virgin olive oil group.

LEfSe analysis used a non-parametric factorial Kruskal-Wallis sum-rank test to determine biomarkers in different groups. As depicted in Figure 4A, LEfSe results showed different biomarkers of four groups. *c__Bacteroidia, o__Bacteroidales,* Firmicutes *__Bacteroidetes, g___Ruminococcus__gauvreauii_group, uncultured_bacterium_g__Ruminococcus__gauvreauii_group, f__Bacteroidaceae, g__Bacteroides, s__uncultured_bacterium_g_Bacteroides, g__Ruminococcaceae_UCG_005, s__uncultured_bacterium_g_Ruminococcaceae_UCG_005, g__Rothia, f__Micrococcaceae, g__uncultured_bacterium_f_Bacteroidales_S24_7_group, s__uncultured_bacterium_f_Bacteroidales_S24_7_group, f__Bacteroidales_S24_7_group, o__Micrococcales, g__Faecalitalea*, and *s__uncultured_bacterium_g_Faecalitalea* were biomarkers of NC group. *f__Lachnospiraceae, g__Lachnoclostridium, s__uncultured_bacterium_g_Lachnoclostridium, p__Actinobacteria, c__Actinobacteria, s__uncultured_bacterium_g__Ruminococcus__torques_group, g___Ruminococcus__torques_group* and *s__Corynebacterium_glutamicum* were biomarkers of M group. *g__Faecalibaculum, s__uncultured_bacterium_g_Faecalibaculum, p__Proteobacteria, g__Ruminococcaceae_UCG_014, s__uncultured_bacterium_g_Ruminococcaceae_UCG_014, g__Klebsiella, s__uncultured_bacterium_g_Clostridium_sensu_stricto_1, f__Clostridiaceae_1, g__Clostridium_sensu_stricto_1* and *f__Peptostreptococcaceae* were biomarkers of HOPO group. *c__Gammaproteobacteria, s__uncultured_bacterium_g_Blautia, o__Enterobacteriales, f__Enterobacteriaceae, s__uncultured_bacterium_g_Allobaculum*, and *g__Allobaculum* were biomarkers of EVOO group.

Metastats analysis was used to screen out the species causing the difference in the composition of the two groups. At family levels, species with a significant difference between M group vs. NC group, HOPO group vs. M group, and EVOO group vs. M group were shown in Figure 4B (*p* < 0.05). There were 23, 9, and 10 species with a significant difference respectively (*p* < 0.05). Compared with NC group, M group had significantly increased *Aerococcaceae, Corynebacteriaceae, Enterococcaceae, Methylobacteriaceae, Microbacteriaceae, Moraxellaceae, Planococcaceae, Staphylococcaceae, Xanthomonadaceae, Brucellaceae, Caulobacteraceae, Deferribacteraceae, Enterobacteriaceae, uncultured_bacterium_o_Gastranaerophilales, Sphingobacteriaceae*, and *Verrucomicrobiaceae* (*p* < 0.05), and significantly decreased *Christensenellaceae, Micrococcaceae*, *Anaeroplasmataceae*, *Bacteroidales_S24-7_group, uncultured_bacterium_o_Mollicutes_RF9, Ruminococcaceae*, and *Family_XIII* (*p* < 0.05). Compared with M group, HOPO group had significantly increased *Clostridiaceae_1, Anaeroplasmataceae, Bifidobacteriaceae, Erysipelotrichaceae*, and *Planococcaceae* (*p* < 0.05), and significantly decreased *Lachnospiraceae, Micrococcaceae, Streptococcaceae*, and *Bacteroidaceae* (*p* < 0.05). Compared with M group, EVOO group had significantly increased *Eubacteriaceae, Bifidobacteriaceae, Anaeroplasmataceae, Erysipelotrichaceae*, and *Clostridiaceae_1* (*p* < 0.05), and significantly decreased *Corynebacteriaceae, Enterococcaceae, Aerococcaceae, Staphylococcaceae*, and *Coriobacteriaceae* (*p* < 0.05).

At genus levels, species with a significant difference between M group vs. NC group, HOPO group vs. M group, and EVOO group vs. M group were shown in Figure 4C (*p* < 0.05). There were 40, 22, and 13 species with a significant difference respectively (*p* < 0.05, the relative abundance >0.1%). Compared with NC group, M group had significantly increased, *Acinetobacter, Bilophila, Aerococcus, Ruminococcaceae_UCG-003, Coprococcus_1, Staphylococcus, uncultured_bacterium_f_Coriobacteriaceae, Klebsiella, Escherichia-Shigella, Acinetobacter, Ruminiclostridium_9, Enterococcus, Roseburia, Corynebacterium_1, [Ruminococcus]_torques_group*, and *Lachnoclostridium* (*p* < 0.05), and significantly decreased *Papillibacter, Candidatus_Soleaferrea, Anaeroplasma, Enterorhabdus, Anaerostipes, [Eubacterium]_ruminantium_group, [Eubacterium]_ruminantium_group, Lachnospiraceae_UCG-008, Christensenellaceae_R-7_group, uncultured_bacterium_o_Mollicutes_RF9, [Eubacterium]_xylanophilum_group, Ruminococcaceae_UCG-009, Family_XIII_AD3011_group, [Eubacterium]_fissicatena_group, [Eubacterium]_nodatum_group, Ruminococcus_2, Sellimonas, [Eubacterium]_coprostanoligenes_group, Ruminococcaceae_UCG-014, Erysipelatoclostridium, Desulfovibrio, Rothia, Faecalitalea, Ruminococcaceae_UCG-005, uncultured_bacterium_f_Bacteroidales_S24-7_group*, and *[Ruminococcus]_gauvreauii_group* (*p* < 0.05). Compared with M group, HOPO group had significantly increased *Olsenella, Peptoclostridium, Ruminococcaceae_UCG-009, Weissella, Bifidobacterium, [Eubacterium]_fissicatena_group, [Eubacterium]_coprostanoligenes_group, Ruminococcaceae_NK4A214_group, Clostridium_sensu_stricto_1, Ruminococcaceae_UCG-014*, and *Faecalibaculum* (*p* < 0.05), and significantly decreased *Bilophila, Leuconostoc, [Eubacterium]_nodatum_group, Lactococcus, uncultured_bacterium_f_Coriobacteriaceae, Streptococcus, Rothia, [Ruminococcus]_torques_group, Bacteroides, Lachnoclostridium*, and *Blautia* (*p* < 0.05). Compared with M group, EVOO group had significantly increased *Olsenella, Bifidobacterium, [Eubacterium]_fissicatena_group, Ruminococcaceae_UCG-014*, and *Allobaculum* (*p* < 0.05), and significantly decreased *Bilophila, Aerococcus, Staphylococcus, uncultured_bacterium_f_Coriobacteriaceae, Faecalitalea, Enterococcus, Corynebacterium_1,* and *[Ruminococcus]_torques_group* (*p* < 0.05). *Bifidobacterium* is one of the most important probiotics in gut, which has many prebiotic functions, such as improving lipid metabolism, regulating immunity, and retarding pathogen, etc. HOPO and EVOO supplementation enriched the relative abundance of *Bifidobacterium* (NC, M, HOPO and EVOO group: 0.090%, 0.079%, 0.52% and 0.23%).

## 4. Discussion

MS has been regarded as one of the urgent worldwide public health problems of this century, which enhances the risk of developing CVD and T2DM [1,26]. Dietary pattern is one of the most important factors in the pathogenesis of MS. The type and quantity of dietary fat have a significant impact on the components of MS [27]. In the present study, we demonstrated that both HOPO and EVOO can attenuate the HFHFD-induced MS, which was associated with reshaping the profile of gut microbiota. The results showed that both HOPO and EVOO significantly reduced body weight gain, and significantly improved insulin sensitivity and HDL/LDL. Moreover, the 16S rRNA gene sequence data indicated that HOPO and EVOO prevented HFHFD-induced gut disorder, and enriched relative abundance of probiotics, such as *Bifidobacterium*.

As one of the core ingredients of Mediterranean diet, olive oil is recognized as a healthy dietary oil, and EVOO has the highest quality among olive oils. The health benefits of EVOO have been attributed not only to the high MUFA content but also to the minor bioactive phytochemicals content. Oleic acid not only has basic nutritional functions, but also has many health functions, such as anti-inflammation, anti-hypertension, cardiovascular disease, and diabetes prevention [28]. Additionally, as a MUFA, oleic acid has strong oxidation stability. Thus, high-oleic acid peanuts have strong oxidation stability, long shelf-life, better nutritional and health functions that are favored by consumers. Compared to olive oil, HOPO has a similar and even higher MUFA level, and is also rich in minor bioactive phytochemicals, such as phytosterols and polyphenol. In fact, as one of the most important oil crops in the world, peanuts are grown on all six continents except Antarctica, and is therefore more widely grown than olives [29]. In the present study, we found that both HOPO and EVOO could improve serum lipids profile in HFHFD-induced MS rats, and protective effects of HOPO were better than EVOO. Due to the high production levels, moderate prices and attractive flavor, HOPO has great potential to be used as a health-promotive oil, especially in the countries and areas unsuitable for olive cultivation.

Accumulating experimental evidence has suggested that the human gut microbiota plays a fundamental role in the metabolic capacity for processing nutrients and host health [30,31,32]. Recent research indicates the possibility that in attenuating MS effects of virgin olive oil the modulation of intestinal microbiota could be involved in [25]. EVOO diet-fed mice had significant lower body weight than butter groups (*p* < 0.01), although EVOO group had a higher food intake level. Serum insulin level in EVOO group was significantly higher than the butter group, while serum glucose level without significant difference between two groups [25]. These results were in line with this study. As expected, supplementation of MUFA-rich oils-HOPO and EVOO could significantly suppress the HFHFD-induced body weight gain, insulin resistance, and gut disorder by modulating the gut microbiota. *Bifidobacterium* is a gram-positive, anaerobe bacterium belonging to the family *Bifidobacteriaceae*, which is one of the most important probiotics in human gut. Relative abundance reduction of *Bifidobacterium* has been demonstrated to be associated with obesity and its related diseases [33]. The possible protective mechanism is maintaining normal intestinal permeability and restraining inflammation caused by lipopolysaccharide. The relative abundance of *Bifidobacterium* in HOPO and EVOO group was significantly higher than M group in this study. The family *Lachnospiraceae* is supposed to facilitate lipopolysaccharide transfer from the intestinal tract to the blood [34]. Previous studies have suggested that *Lachnospiraceae* family is significantly increased in both obese nonalcoholic fatty liver disease and T2DM in humans [35,36]. *Blautia* is a genus of the family *Lachnospiraceae*, which was thought to take part in the development of glucose metabolism disturbances [37]. In this study, relative abundance of *Lachnospiraceae* and *Blautia* in HOPO group and EVOO group was lower than M group, but only HOPO group with significant difference.

However, there are some gut microbiota results which are unlike some other studies. Many studies have shown that hosts with obesity, T2DM and high fat diet has a higher F/B ratio [15,38,39]. The imbalance of F/B ratio might be a contributing factor to obesity and its relative metabolic disease. In this study, F/B ratio of M group was increased compared to NC group, but F/B ratio of HOPO group and EVOO group was higher than M group. A few studies indicated that F/B ratio was not necessarily related with obesity linked diseases [40]. Duncan et al. found that there is no relationship between BMI or absolute weight loss and the relative proportions of Bacteroidetes and Firmicutes of colonic bacteria in obese or non-obese subjects [41]. This correlation is controversial and needs further research so far. Moreover, *Akkermansia* be regarded as one of major probiotics which can increase the thickness of intestinal mucin and improve the barrier function of intestinal mucosa, so as to inhibit the obesity caused by high-fat diet [42]. However, relative abundance of *Akkermansia* in NC group was significantly lower than M group, and there was no significant difference between HOPO group vs. M group and EVOO group vs, M group. The variation on *Akkermansia* abundance of the same dietary intervention may depend on its baseline abundance level. Subjects in the caloric restricted diet group with a high baseline level of *Akkermansia* had a decrease in abundance of *Akkermansia,* while there was an increase in subjects with a low baseline level [7]. The abundance changes of some specific species should not be simply judged as a possible mechanism by the past similar studies. Therefore, we are enlightened that the interactions within gut microbiota species and between gut microbiota and external factors are still insufficiently studied. Above all, we suppose that *Bifidobacterium* is the key probiotic of rats fed with HOPO and EVOO. Lower *Lachnospiraceae* and *Blautia* relative abundance were also related to modulation of the gut microbiota.

## 5. Conclusions

In conclusion, the results show that both HOPO and EVOO can prevent HFHFD-induced MS associated with modulation of the gut microbiota. HOPO and EVOO significantly increased *β*-diversity of gut microbiota composition. The gut microbiota profile of HOPO group and EVOO group and NC group were more similar than NC group to M group, which means HOPO and EVOO could relieve gut microbiota disorder. Furthermore, some probiotic microbiotas were modulated by HOPO and EVOO, such as *Bifidobacterium*. The structure of glyceryl monooleate, fatty acid profile, and bioactive phytochemicals may result in some differences of plasm parameters and specific gut microbiota species. Our findings provide the experimental evidence to use HOPO for MS prevention, and offer a new insight into the MS prevention effect of high oleic acid oils from the perspective of regulating intestinal flora. Further investigations are necessary to establish the precise relationship in human between MS and HOPO consumption.

## Figures and Tables

**Table 1:** Effects of treatments on biomarkers of blood glucose level and insulin resistance.

	NC	M	HOPO	EVOO
FBG (mmol/L)	4.38 ± 0.42 ^a^	6.08 ± 0.76 ^bc^	6.53 ± 0.65 ^c^	5.79 ± 0.56 ^b^
OGTT AUC				
0 weeks	842.75 ± 77.82 ^a^	879.81 ± 70.11 ^a^	874.69 ± 46.69 ^a^	862.56 ± 73.47 ^a^
4 weeks	951.63 ± 80.58 ^a^	1070.88 ± 109.16 ^b^	1072.50 ± 33.43 ^b^	1052.81 ± 98.91 ^b^
8 weeks	914.06 ± 62.62 ^a^	983.63 ± 108.81 ^ab^	957.50 ± 59.60 ^ab^	1005.56 ± 121.55 ^b^
12 weeks	892.19 ± 110.37 ^a^	972.13 ± 96.69 ^a^	972.56 ± 82.52 ^a^	959.75 ± 100.30 ^a^
ITT AUC				
0 weeks	408.69 ± 32.14 ^a^	414.56 ± 38.97 ^a^	433.69 ± 30.55 ^a^	402.94 ± 51.52 ^a^
4 weeks	417.69 ± 98.06 ^a^	524.13 ± 61.95 ^b^	544.19 ± 80.49 ^b^	506.00 ± 80.16 ^b^
8 weeks	389.31 ± 64.06 ^a^	532.31 ± 69.35 ^c^	470.81 ± 44.06 ^b^	479.94 ± 55.97 ^b^
12 weeks	393.56 ± 71.98 ^a^	523.19 ± 83.27 ^b^	425.56 ± 87.93 ^a^	407.56 ± 67.30 ^a^
Insulin (ug/L)	0.89 ± 0.12 ^a^	1.70 ± 0.33 ^c^	1.19 ± 0.21 ^b^	1.36 ± 0.26 ^b^
HOMA-IR	4.15 ± 0.73 ^a^	10.88 ± 1.71 ^c^	8.67 ± 2.02 ^b^	8.43 ± 1.86 ^b^

Data are expressed as means ± SD (*n* = 12 for each group). The different letters in same line represent significant differences between different groups (*p* < 0.05).

**Table 2:** Effects of treatments on biomarkers of metabolic syndrome in serum.

	NC	M	HOPO	EVOO
TC (mmol/L)	3.00 ± 0.79 ^a^	3.62 ± 0.50 ^b^	3.06 ± 0.65 ^a^	3.18 ± 0.40 ^ab^
TG (mmol/L)	1.95 ± 0.42 ^a^	5.99 ± 2.09 ^c^	4.23 ± 1.45 ^b^	5.79 ± 2.22 ^c^
HDL (mmol/L)	0.84 ± 0.15 ^a^	0.71 ± 0.25 ^a^	0.69 ± 0.23 ^a^	0.76 ± 0.25 ^a^
LDL (mmol/L)	0.40 ± 0.14 ^a^	0.56 ± 0.21 ^b^	0.38 ± 0.13 ^a^	0.50 ± 0.18 ^ab^
HDL/LDL	2.10 ± 0.25 ^c^	1.27 ± 0.12 ^a^	1.82 ± 0.18 ^b^	1.52 ± 0.21 ^b^
FFA (umol/L)	459.96 ± 38.76 ^a^	555.80 ± 55.34 ^b^	526.80 ± 60.18 ^b^	512.06 ± 47.15 ^b^
TNF-a (ug/mL)	196.11 ± 34.67 ^a^	257.74 ± 75.73 ^b^	208.16 ± 58.73 ^ab^	220.35 ± 63.03 ^ab^

Data are expressed as means ± SD (*n* = 12 for each group). The different letters in same line represent significant differences between different groups (*p* < 0.05).

## Supplementary Materials

The following are available online at https://www.mdpi.com/2072-6643/11/12/3005/s1, Figure S1: Venn diagram of operational taxonomic units abundance, Table S1: Fatty acid profiles of HOPO and EVOO, Table S2: Compositions of the diets, Table S3: Composite specific bacterial primers, Table S4: Simpson, Shannon, ACE and Chao1.
