# Supplementary material for: High Oleic Acid Peanut Oil and Extra Virgin Olive Oil Supplementation Attenuate Metabolic Syndrome in Rats by Modulating the Gut Microbiota
Source: Nutrients. 2019 Dec 7;11(12):3005. doi: 10.3390/nu11123005 (PMC6950752; doi:10.3390/nu11123005)
Supplement: Supplementary file 1 [file nutrients-11-03005-s001.pdf]

Supplementary materials

Table S1. Fatty acid profiles of HOPO and EVOO.

Table S2. Compositions of the diets.

Table S3. Composite specific bacterial primers

Table S4. Simpson, Shannon, ACE and Chao1.

Figure S1. Venn diagram of operational taxonomic units abundance.

Table S1. Fatty acid profiles of HOPO and EVOO. HOPO: high-oleic acid peanut oil, EVOO: extra virgin olive oil.

Fatty acid / %	HOPO	EVOO
UFA	9.66	14.30
MUFA	77.23	78.48
PUFA	10.10	8.41

Table S2. Compositions of the diets. NC: normal control group, M: model group, HOPO: high-oleic acid peanut oil group, EVOO: extra virgin olive oil group.

	NC	M	HOPO	EVOO
Fat / % kcal	10	45	45	45
Protein / % kcal	14.1	14.1	14.1	14.1
Carbohydrate / % kcal	75.9	40.9	40.9	40.9
HOPO / %	—	—	10	—
EVOO / %	—	—	—	10
Soybean oil / %	4	3	—	—
Lard / %	—	19.6	12.6	12.6
Energy	3.6 kcal/g	4.5 kcal/g	4.5 kcal/g	4.5 kcal/g

Table S3. Composite specific bacterial primers

Target gene	Sequence(5'-3')	Product Length
Real-time primers		
ACC-1	F:GGACAGACTGATCGCAGAGA	75
	R:TGGAGAGCCCCACACACA	
CD36	F:GGAACTGTGGGCTCATTGC	68
	R:CATGAGAATGCCTCCAAACAC	
CPT1β	F:GGCACCTCTTCTGCCTTTAC	85
	R:TTTGGGTCAAACATGCAGAT	
FABP	F:AGCATCATAACCCTAGATGGCG	114
	R:CATAACACATTCCACCACCAGC	
FAS	F:CCCTTGATGAAGAGGGATCA	115
	R:ACTCCACAGGTGGGAACAAG	
PGC1α	F:GGAGCCGTGACCACTGACA	176
	R:TGGTTTGCTGCATGGTTCTG	
UCP1	F:TAAGCCGGCTGAGATCTTGT	84
	R:GGCCTCTACGACTCAGTCCA	
UCP3	F:ATGAGTTTTGCCTCCATTCG	184
	R:GGCGTATCATGGCTTGAAAT	
GAPDH	F:GTCGTGGATCTGACGTGCC	72
	R:TGCCTGCTTCACCACCTTCT	
16S rRNA (V3+V4) primers		
	F:ACTCCTACGGGAGGCAGCA	
	R:GGACTACHVGGGTWTCTAAT	

Figure S1. Venn diagram of operational taxonomic units abundance. NC: normal control group, M: model group, HOPO: high-oleic acid peanut oil group, EVOO: extra virgin olive oil group.

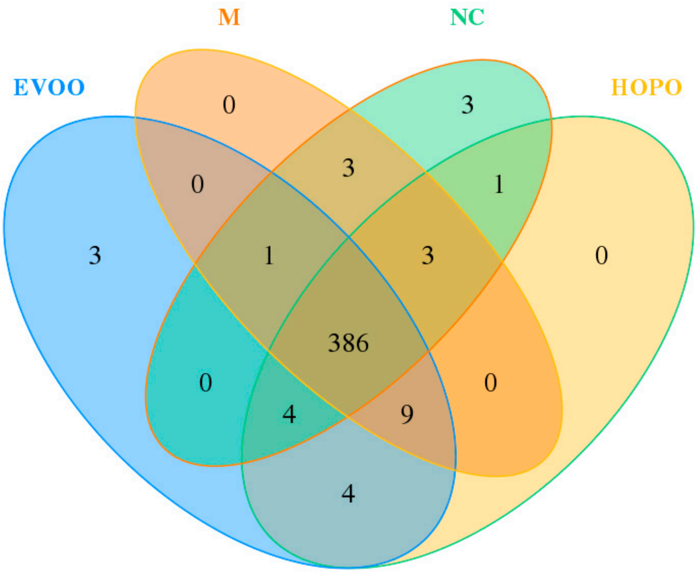

Table S4. Simpson, Shannon, ACE and Chao1. NC: normal control group, M: model group, HOPO: high-oleic acid peanut oil group, EVOO: extra virgin olive oil group.

	NC	M	HOPO	EVOO
Simpson	0.044±0.011	0.043±0.012	0.053±0.034	0.054±0.019
Shannon	4.00±0.17	3.95±0.22	3.91±0.41	3.81±0.31
ACE	343.87±12.01a	347.95±18.74a	356.04±17.66b	369.23±14.60ab
Chao1	348.54±9.54a	351.29±19.03a	361.13±22.94ab	374.92±20.01b

Data are expressed as means ±SD. The different letters represent significant differences between different groups ($p < 0.05$).
